# Increased frequency of Mediterranean fever gene variants in multiple myeloma

**DOI:** 10.3892/ol.2014.2407

**Published:** 2014-08-04

**Authors:** SERKAN CELIK, FATIH TANGI, CAGATAY OKTENLI

**Affiliations:** 1 Division of Oncology, GATA Haydarpasa Training Hospital, Istanbul, Turkey; 2 Division of Internal Medicine, GATA Haydarpasa Training Hospital, Istanbul, Turkey; 3 Department of Internal Medicine, Anadolu Medical Center, Kocaeli, Turkey

**Keywords:** *MEFV* gene, multiple myeloma, nuclear factor-κB, interleukin-1β

## Abstract

High frequencies of inherited variants in the Mediterranean fever (*MEFV)* gene have been identified in patients with multiple myeloma (MM). The sample size of the present pilot study was small, therefore, the actual frequency of inherited variants in the *MEFV* gene could be investigated in patients with MM. Twenty-eight patients with MM and 65 healthy controls were included in the study. Six heterozygous and one homozygous (E148Q/E148Q) variant was identified in patients with MM. None of the patients had a family history compatible with familial Mediterranean fever. In the healthy control group, 11 heterozygous variants were identified. The difference in the overall frequency of the inherited variants in the *MEFV* gene between the MM patients and the controls was statistically significant (χ^2^=4.905; P=0.027). In conclusion, a high frequency of inherited variants in the *MEFV* gene was identified in patients with MM. Based on the current data, it is hypothesized that the *MEFV* gene is a cancer susceptibility gene. Additional evidence, such as familial aggregation, monozygotic versus dizygotic twin concordance, and tumors in genetically engineered model organisms, is required in order to support this hypothesis.

## Introduction

Familial Mediterranean Fever (FMF) is the most common Mendelian autoinflammatory disorder, which is characterized by recurrent attacks of fever with peritoneal, pleural or synovial inflammation (1–4). Missense mutations in the Mediterranean fever (*MEFV)* gene have been shown to be causative of the disease (5). The 781-aa protein product of *MEFV,* denoted pyrin (also known as marenostrin) is produced in neutrophils, dendritic cells, eosinophils, monocytes, and synovial fibroblasts (6–10). Currently, >270 inherited variants and polymorphisms in *MEFV* have been reported in the Infevers Database (http://fmf.igh.cnrs.fr/ISSAID/infevers). Most of the inherited variants in *MEFV* are located in exon 2 and 10 of the transcript (11). Pyrin contains several domains, including a pyrin domain (12–14), which is involved in homotypic protein-protein interactions in inflammatory and apoptotic signaling pathways (7,15). Although the underlying mechanism is still being investigated, pyrin potentially plays a role in the modulation of interleukin-1β (IL-1β) and nuclear factor-κB (NF-κB) (16–20). Any inherited variants in the *MEFV* gene may cause inflammation and apoptosis due to the altered control of pyrin in the activation of IL-1β and NF-κB (21,22).

Multiple myeloma (MM) is a neoplastic plasma-cell disorder that is characterized by the aberrant expansion of monotypic plasma cells within the bone marrow (23,24). Perturbed signaling pathways that control normal physiological processes, and mutations in several protooncogenes and tumor suppressor genes, can lead to the development of MM (23). Although NF-κB functions in the pathogenesis of MM (25,26), whether inherited variants in MEFV can lead to constitutive NF-κB activation and cause a tendency for MM remains to be determine. Accumulated evidence has shown that there is a high frequency of inherited variants in *MEFV* in patients with hematological malignancies as compared with the general population (27–31). This association has been included in the Genetic Association Database, Record 704091 (http://geneticassociationdb.nih.gov/cgi-bin/view.cgi?table=allview&id=704091). In one of our previous studies, an increased frequency of inherited variants in *MEFV* in patients with MM was observed (28). Since the sample size was small in the present pilot study, the actual frequency of inherited variants in *MEFV* in patients with MM was able to be investigated.

## Materials and methods

### Subjects

Twenty-eight (17 male and 11 female) patients with MM and 65 healthy controls (40 male and 25 female) were included in the study. FMF patients or subjects who had a family history of FMF were excluded. The study protocol conformed to the ethical guidelines of the Helsinki Declaration. Informed consent was obtained from all patients and controls. The local Ethics Committee and Institutional Review Board approved the study. All the patients donated 2 ml of blood, collected in an ethylenediaminetetraacetic acid tube. The eight inherited variants in the *MEFV* gene (M694I, M694V, M680I (G/C-A), V726A, R761H, E148Q and P369S) were detected using the Dr. Zeydanli^®^ FMF Type I PCR system (Ankara, Turkey) 5′ nuclease assay method using an ABI 7500 (Applied Biosystems, Foster City, CA, USA) quantitative polymerase chain reaction system, as previously reported (27).

### Statistical analysis

Data were analyzed using SPSS 17.0 (SPSS Inc., Chicago, IL, USA) statistical software. Differences between the groups were analyzed using a χ^2^ test. P≤0.05 was considered to indicate a statistically significant difference.

## Results

The mean age of the patients with MM and healthy controls was 59.38±22.88 years (age range, 32–84) and 30.25±10.62 years (age range, 20–45), respectively. Hematological characteristics and identified *MEFV* gene variants in patients with MM are shown in Table I. Six heterozygous and one homozygous (E148Q/E148Q) variant in patients with MM was identified. None of the subjects had a family history compatible with FMF. In the healthy control group, 11 heterozygous variants were identified. M680I, M694I and R761H inherited *MEFV* gene variants were not found in any of the groups. The P369S variant was found in one healthy control.

Analytical data concerning the overall inherited *MEFV* variant frequency between patients with MM and comparisons with healthy controls are given in Table II. The difference in the overall frequency of the inherited variants in the *MEFV* gene between MM patients and the controls was statistically significant (χ^2^=4.905; P=0.027). When the distribution was compared between the patients and the controls, the frequency of the E148Q variant was significantly higher in the patient group as compared with the controls (χ^2^=7.438; P=0.006), while the M694V was significantly higher in the control group than MM patients (χ^2^=5.658; P=0.017).

## Discussion

In the current study, a high frequency of inherited variants in the *MEFV* gene was identified in patients with MM as compared with the healthy controls. These results are in concordance with our previous study (28). Of note, E148Q is the predominant inherited *MEFV* variant in patients with MM. Pyrin, the protein product of the *MEFV* gene, functions in the modulation of IL-1β and NF-κB. Since IL-1β is important for the anti-tumor immune response, it has been speculated that genetic variations that modify the expression of IL-1β may influence the risk of MM (32). NF-κB is another important transcription factor for the expression of genes critical for tumor promotion, cell proliferation, inflammation, metastasis, angiogenesis, and suppression of apoptosis (33). The function of NF-κB in lymphopoiesis is well recognized and it is an important factor for the regulation of cellular homeostasis of T and B lymphocytes (34–36). Altered NF-κB activation may cause an increased production of cell cycle regulatory and antiapoptotic proteins and may contribute to the abnormal proliferation and survival of neoplastic cells (37–39). It has been reported that the NF-κB signaling pathway is critical in myeloma cell proliferation and the inhibition of apoptosis (40). Furthermore, constitutive nuclear NF-κB activity has been reported in numerous human MM cell lines and primary myeloma cells (41,42). Specifically, as a distinct mechanism, constitutive activation in the NF-κB signaling pathway or blockade of IL-1β secretion due to defective pyrin may be associated with an increased frequency of inherited *MEFV* variants in patients with MM.

The present study has some limitations. Firstly, only eight inherited variants in the *MEFV* gene were screened in the patients. Rare or novel variants therefore have not been identified. Secondly, the family members of the patients included in this study were not screened for the inherited *MEFV* variants. However, the individual and family history for FMF manifestations was negative in these subjects.

In conclusion, a high frequency of inherited *MEFV* gene variants was shown to be associated with MM. Based on the current data, it may be hypothesized that the *MEFV* gene is a cancer susceptibility gene. Additional evidence, such as familial aggregation, monozygotic versus dizygotic twin concordance and analysis of tumors in genetically-engineered model organisms, is required in future studies.

## Figures and Tables

**Table I:** Hematological characteristics and distribution of inherited variants in the Mediterranean fever gene in patients with multiple myeloma.

Patient no.	Age	Gender	Bone marrow plasmacytosis (%)	WBC (x10^9^/l)	PLTS (x10^9^/l)	Hb (g/dl)	ESR (mm/h)	Inherited variants in *MEFV* gene
1	78	M	65	4,050	104	10	102	E148Q/Unknown^a^
2	54	M	90	93,700	2,28	8,7	91	E148Q/Unknown
3	70	M	2	5,980	240	12,4	46	E148Q/Unknown
4	65	M	35	31,200	156	9,4	92	E148Q/E148Q
5	74	F	40	29,300	111	13,9	91	V726A/Unknown
6	49	F	45	7,660	218	15	135	M694V/Unknown
7	68	F	40	4,440	58,200	9,3	60	E148Q/Unknown

a Unknown variant indicates that the chromosome carries a mutation not determined in our study.

F, female; M, male; WHO, World Health Organization classification; Hb, hemoglobin; WBC, white blood cells; PLTS, platelets; ESR, erythrocyte sedimentation rate; MEFV, Mediterranean fever.

**Table II:** Comparison of the inherited variant frequency in the Mediterranean fever gene between patients with multiple myeloma and normal controls.

			Heterozygote variant frequencies in MEFV gene							
			
Variables	n	Overall inherited variant frequency in MEFV gene	Homozygote (E148Q/E148Q)	M694V/Unknown^a^	E148Q/Unknown	M680I/Unknown	V726A/Unknown	M694I/Unknown	R761H/Unknown	P369S/Unknown
Normal controls	65	0.084	0	0.038	0.015	0	0.023	0	0	0.007
Multiple myeloma	28	0.143	1	0.017	0.107	0	0.017	0	0	0
χ^2^		4.905	-	5.658	7.438		0.535	-	-	0.433
P-value		0.027	-	0.017	0.006	-	NS	-	-	NS

a Unknown variant indicates that the chromosome carries a mutation not determined in our study;

NS, non-significant; MEFV, Mediterranean fever.
